# On the Impact of Anomalous Noise Events on Road Traffic Noise Mapping in Urban and Suburban Environments

**DOI:** 10.3390/ijerph15010013

**Published:** 2017-12-23

**Authors:** Ferran Orga, Francesc Alías, Rosa Ma Alsina-Pagès

**Affiliations:** GTM-Grup de recerca en Tecnologies Mèdia, La Salle-Universitat Ramon Llull, C/Quatre Camins, 30, 08022 Barcelona, Spain; falias@salleurl.edu (F.A.); ralsina@salleurl.edu (R.M.A.-P.)

**Keywords:** anomalous noise event, wireless acoustic sensor network, noise pollution, *L_Aeq_*, acoustic impact, road traffic noise, noise map, health effects

## Abstract

Noise pollution is a critical factor affecting public health, the relationship between road traffic noise (RTN) and several diseases in urban areas being especially disturbing. The Environmental Noise Directive 2002/49/EC and the CNOSSOS-EU framework are the main instruments of the European Union to identify and combat noise pollution, requiring Member States to compose and publish noise maps and noise management action plans every five years. Nowadays, the noise maps are starting to be tailored by means of Wireless Acoustic Sensor Networks (WASN). In order to exclusively monitor the impact of RTN on the well-being of citizens through WASN-based approaches, those noise sources unrelated to RTN denoted as Anomalous Noise Events (ANEs) should be removed from the noise map generation. This paper introduces an analysis methodology considering both Signal-to-Noise Ratio (SNR) and duration of ANEs to evaluate their impact on the A-weighted equivalent RTN level calculation for different integration times. The experiments conducted on 9 h of real-life data from the WASN-based DYNAMAP project show that both individual high-impact events and aggregated medium-impact events bias significantly the equivalent noise levels of the RTN map, making any derived study about public health impact inaccurate.

## 1. Introduction

Environmental noise pollution is increasing year after year because of population growth and the consequent expansion of transportation systems, including highways, railways and airways [1]. It is not merely an annoyance, since several studies warn about its adverse effects on people pointing to health-related problems [2]. Most of the conducted studies address the effects of long-term exposure to environmental noise, mainly focused on concentration issues, sleep disturbance and stress [3], emphasizing the negative effects on children [4].

Research also specifically analyzes the association between road traffic noise and several diseases in suburban and urban areas. Öhrström states that the influence of road-traffic noise implies an increase in tiredness and disturbs the sleep [5]. In addition, Botteldooren et al. analyze the influence of road traffic on noise annoyance in neighborhoods [6], while Jakovljevic et al. conclude that the most significant noise source in urban areas is road traffic noise, according to the interviewed residents [7].

In order to ensure that these studies only evaluate the impact of road traffic noise (RTN) on the well-being of citizens, those noise sources unrelated to RTN should be removed from the study as they could alter the conclusions significantly. This is usually assured by the experts conducting the acoustic measurements.

The European Union has reacted to this alarming increase of environmental noise pollution, especially in large agglomerations, approving the Environmental Noise Directive 2002/49/EC (END) [8]. In accordance with the END, the CNOSSOS-EU methodological framework pretends to improve the consistency and comparability of noise assessment results across the EU Member States [9]. The main pillars of the END are the following: (i) determining the noise exposure, (ii) making the updated information related to noise available to citizens, and (iii) preventing and reducing the environmental noise where necessary. Moreover, the END requires the European Member States to publish noise maps and action plans for agglomerations with more than 100,000 inhabitants and major roads, railways and airports every five years, and introduces the need to discern between the different sound sources [8].

Recent technological advances have posed a significant change of paradigm to address the END regulatory requirements, mainly based on the design and development of Wireless Acoustic Sensor Networks (WASNs). Authors have suggested their WASN-based solutions involving cities as Barcelona (Spain) [10] and Pisa (Italy) [11]. However, most of these projects do not identify the noise typology of the area of interest, thus addressing noise monitoring in a holistic way. As an exception, in [12], sound recognition is applied together with a subjective survey in order to cross both acoustic and subjective perception of noise sources.

Nevertheless, since the identification of sound sources is conducted after computing the noise levels, this information cannot be used to modify the noise map calculation accordingly. To this aim, the DYNAMAP project [13] pretends to deploy a WASN to tailor dynamic noise maps of RTN in suburban and urban areas [13]. In an attempt to monitor only the RTN, those events unrelated to RTN denoted as Anomalous Noise Events (ANEs) (e.g., birds, people, sirens, etc.) have to be detected and removed automatically before computing the A-weighted equivalent noise levels ($L_{Aeq}$) of RTN so as to obtain a reliable picture of citizens’ exposure to this pollutant [14]. In addition, the contribution of these ANEs to the $L_{Aeq}$ calculation should be studied in order to obtain reliable RTN maps, from which accurate studies of the impact of the RTN on health can be derived from WASN-based approaches.

The goal of this paper is to evaluate the impact of the ANEs on the computation of RTN $L_{Aeq}$ in real-life suburban and urban scenarios. The datasets contain 17 recording locations in total, defined in [15] with the purpose of capturing real-life ANE diversity and sampling correctly both pilot areas. To that effect, we introduce an analysis methodology that takes into account the duration and Signal-to-Noise Ratio (SNR) of the ANEs with respect to the surrounding RTN levels. A priori, it seems reasonable that the higher the duration and the SNR of an individual ANE, the higher the impact on the final $L_{Aeq}$, but the importance of the impact of an ANE with short duration and high SNR, or ANE with long duration and low SNR is not so foreseeable. For this reason, a joint study of duration and SNR is also conducted considering the impact of individual ANEs on the $L_{Aeq}$ computation for different integration times, besides evaluating their aggregated impact for a given integration span. integration times, and validating the impact for several aggregated ANEs in a one-time integration span.

## 2. Related Work

In this section, first, we review several representative investigations focused on the study of the impact noise to health, together with those projects focused on measuring the quality of life of citizens due to road traffic noise. Second, we describe different approaches to address automatic noise monitoring by means of low-cost WASN and the use of their measurements to tailor noise maps.

### 2.1. Influence of Noise on Public Health

Some studies warn about the noise exposure of citizens in certain cities, with some critical examples as the case of Tainan (Taiwan). According to [16], over 90% of Tainan City inhabitants are exposed to unacceptable noise levels—62 dB(A), as defined by US Department of Housing and Urban Development. In addition, 93.3% of the inhabitants of Curitiba (Brazil) are exposed to a sound level over 65 dB(A), as stated by [17]. According to [18], 90% of the population of Cáceres (Spain) is also affected by a sound level of 65 dB(A) during working time. The reader may find other examples related to sound exposure in the aforementioned countries seeing the cited references. Other cases include India [19] and China [20].

The World Health Organization (WHO) quantifies the healthy life years lost in Europe in terms of “Disability-Adjusted Life-Years” (DALYs) [21], concluding that the diseases related to noise exposure produce a loss of a million healthy life years in western Europe every year. Furthermore, research has found that noise exposition does not only affect health, but also social and economic aspects [1]. In addition, a permanent hearing loss and possible tinnitus is associated with noise exposure, as stated in [22], especially caused by a prolonged listening of loud music through PLDs (Personal Listening Devices). Another relation between environmental noise and adverse birth outcomes can be found in [23], concluding that a low quality association exists between aircraft noise and preterm birth. Furthermore, other studies connect the noise pollution to mental illnesses [24], diabetes [25] and other heart diseases [26].

Moreover, some authors remark on the importance of road traffic noise to the living quality of the neighborhoods, conducting several subjective studies: Botteldooren et al. compare a set of indicators related to sound exposure in [6], Van Renterghem et al. conclude the importance of having a quiet facade in the dwellings [27]. In addition, RTN increased the tiredness and disturbs the sleep, says Öhrström in [5], who even states that access to quiet parts of the residence contributes to physiological and psychological well-being. Finally, a study by Jakovljevic et al. determines the principal factors for high noise annoyance in adults using several indicators, as stress scores, age and other location variables (e.g., orientation of the rooms towards the street). After conducting perception surveys, most of the interviewed residents found road traffic the most significant noise source. A review of the transport noise interventions and their impacts on health is conducted in [28], studying the European region in particular.

### 2.2. Noise Monitoring

In order to satisfy the increasing demand of automatically monitoring the noise levels in urban areas, several WASN-based projects are being developed in different countries. The DREAMSys project monitors several UK areas with a distributed sensor network [29]. Other projects pretend to monitor also the urban noise in real-time, as the UrbanSense project in Canada [30], which also pretends to monitor other pollutants as carbon dioxide (CO_2_) and carbon monoxide (CO). The Senseable project in Pisa is based on the concept of real-time city and smart city to measure the sound level in several points [11]. In addition, a noise monitoring network is also being deployed in Barcelona in order to manage the resources efficiently and to reduce the impact of urban infrastructure on the environment [10] and, recently, in Monza, by a LIFE project that implements also a low-cost system [31]. Finally, some projects focus in other areas, such as, for example, highways. In [32], five points along the National Highway of Burdwan have been monitored with an audiometer in order to register the acoustic equivalent level ($L_{eq}$) and carrying a statistical analysis. In addition, the location strategy to evaluate multiple noise sources has been studied in [33,34].

Other noise monitoring projects take into account other data further than the noise equivalent level. In the Smart Sound Monitoring project, De Coensel et al. conducted a study in [12] that crosses acoustic information with subjective perception surveys, allowing for considering the typology of the acoustic information. Furthermore, a sound recognition system is applied in order to give information about the detected sounds and establish a relation between the identified events and the perception surveys. However, the system identifies events only to give information but not to remove these sounds from the noise map.

The aforementioned projects pretend to monitor the noise in determined areas using a low-cost sensors network; however, as far as we know, none of them intend to remove the anomalous events biasing the traffic noise map measurement. With this purpose, the DYNAMAP project pretends to monitor road traffic noise in suburban and urban areas reliably, after removing the anomalous noise events from the road traffic noise map computation [13].

## 3. Analysis Methodology

This section describes the methodology applied to analyze the impact of the ANEs on the equivalent noise level computation of road traffic noise, which details the ANE parametrization and the calculation of their acoustic impact for a certain integration time.

**Figure 1:**
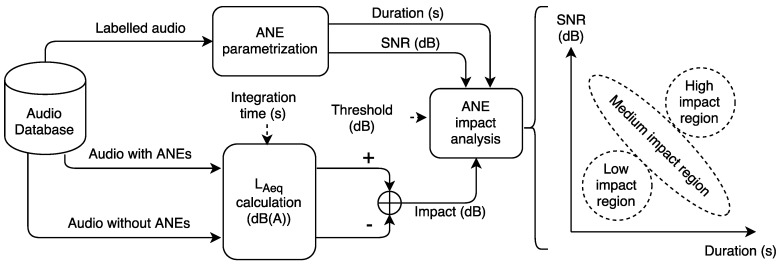
Block diagram of the methodology used to analyze the impact contribution of the Anomalous Noise Events (ANEs) to the $L_{Aeq}$ considering the Signal-to-Noise Ratio (SNR) and the duration of the anomalous event. The analysis includes three regions: (**a**) high-impact, due to ANEs with high SNR and long duration; (**b**) low-impact, due to ANEs with low SNR and short duration; and (**c**) medium impact, which integrates those ANEs falling in between the previous two regions.

Figure 1 depicts the proposed analysis methodology used to determine the impact of a particular ANE in the equivalent noise level computation. The ANE is parametrized by means of their SNR and duration. The analysis takes the raw acoustic data corresponding to an integration time, and computes both the $L_{Aeq}$ considering the ANE and after removing the event. For the latter, the ANE is replaced with a linear interpolation connecting the previous RTN sample with the following. It is worth mentioning that the A-weighted filter is applied before the $L_{eq}$ calculation in an effort to account for the relative loudness perceived by the human ear. After that, the particular impact of an ANE is calculated by deducting the $L_{Aeq}$ of the interpolated ANE from the $L_{Aeq}$ calculation of the entire piece of audio, for a given integration time. The last stage of the analysis methodology associates the impact of the ANE under study with the corresponding SNR and duration. The high-impact ANEs are defined as the ones surpassing the predefined limit.

The impact analysis consists in validating the hypothesis, which states that long ANEs with high-SNR would entail high impact on the $L_{Aeq}$ computation, while short and low-SNR ANEs would affect less in the impact for a given integration time. However, a wide variety of events may be captured from real-life data, including ANEs presenting different possible combinations of duration and SNR and making it possible that long events with low SNR could have a similar impact than those short events with high SNR. To this aim, the study has to be completed by evaluating the aggregated impact of the ANEs for a specific integration time, considering their SNR and their duration. As a consequence, the aggregated impact of several ANEs is expected to be higher than any of their individual impacts by itself, even coming from the low or medium impact region of Figure 1, which could entail the removal from the $L_{Aeq}$ computation of individual ANEs with individual moderate SNR and/or duration.

In the following sections, we describe the main elements of the introduced analysis methodology.

### 3.1. ANE Parametrization

A set of parameters have been defined in order to analyze the impact of the ANEs in detail. Both duration and SNR are considered to analyze the impact of ANEs in the $L_{Aeq}$ computation. The SNR considers the energy of the event in relation to the surroundings; however, it does not take into consideration the duration of the event, which could also impact the $L_{Aeq}$. By considering both parameters, we derive the hypothesis depicted in Figure 1.

- **SNR Calculation.**
  First, the SNR of an ANE is defined as a classical Signal-to-Noise Ratio, but considering that the RTN noise is not stationary. It is evaluated in order to obtain the impact of a particular ANE in relation to the surrounding RTN signal level. The acoustic power of the ANE with respect to the surrounding RTN is calculated as expressed by Equation (1):
  (1) $$P_{x}=\sum_{t=1}^{N}\left(\frac{x{\left(t\right)}^{2}}{N}\right),$$
  where *N* is the number of samples and x(t) is the recorded audio during a certain integration period.
  After the powers of the ANE and the RTN are evaluated, the SNR is calculated as follows:
  (2) $$SNR=10\;log_{10}\left(\frac{P_{ANE}}{P_{RTN}}\right),$$
  where $P_{ANE}$ belongs to the anomalous event and $P_{RTN}$ belongs to the power of $RTN_{1}$ and $RTN_{2}$, the former is the previous RTN to the ANE and the latter is the next portion of RTN sound.
  Due to the relative nature of the measure, it is worth mentioning that the SNR of the event could result in a negative value if the energy of the ANE is less than the previous and following sound. This is normally caused by events that can only be heard in moments when the road traffic noise decreases. In addition, to compute the SNR, previous and following samples to the event are considered; thus, this calculation is only meant for analysis and it is not applicable for real-time decision purposes due to the need of future samples of the signal.
- **Duration of the ANE.**
  The duration of the events is an important factor, since not only the salience of the event is important in what concerns its impact on the $L_{Aeq}$ computation, but also the time the anomalous situation lasts. The duration of the ANE is obtained by means of the difference between the time stamp of the first and the last sample of the labelled dataset. As published in the previous study of the dataset, the duration of the ANEs may vary depending on the typology of the sound and the circumstances of the location [15]. One of the shorter samples observed corresponds to 40 ms length in Rome, and the longer 53 s in Milan, with 0.6 s being the mean of the duration of the ANE.

**Figure 2:**
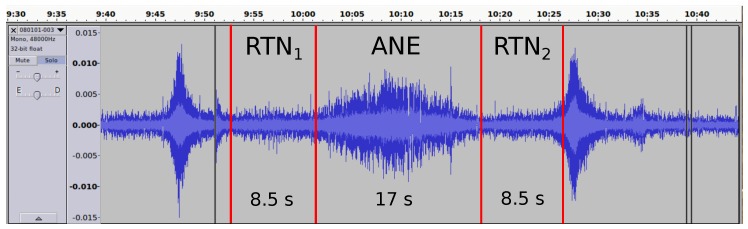
An example of the SNR and duration measurement of a raw acoustic signal corresponding to a 17 s long train ANE. The ANE presents a SNR of 6.6 dB, resulting in an impact of 1.1 dB when the $L_{A5min}$ is calculated, and uses a 5 min time interval evaluation. The left and right RTN regions used to calculate the SNR are marked as $RTN_{1}$ and $RTN_{2}$, respectively.

Figure 2 shows a piece of audio where the SNR and the duration of an ANE has been measured, with the tagged RTN and ANE used to evaluate the SNR, for illustrative purposes.

### 3.2. Impact Calculation

The impact calculation takes into account the integration period of the recording in order to obtain the repercussions of the analyzed event or set of events on the $L_{Aeq}$ computation. It is computed as the difference between the $L_{Aeq}$ considering the individual ANE and the $L_{Aeq}$ replacing the event with the linear interpolation from the last and the following RTN sample of the original raw data. For this reason, in this work, it is referred to as $\Delta L_{Aeq}$.

As a guideline for the evaluation of the impact over the $L_{Aeq}$, and, according to the European Commission Working Group, the maximum tolerated change is 2 dB for a given segment [35]. Therefore, a 2 dB threshold is considered in this work to discern high-impact from low-impact ANEs. The analysis can be conducted for several integration times in order to observe the homogeneity of the ANE impact results; the most critical ANE appear as so in all integration times, despite having different impact values. Thus, the ANE evaluation sorted depending on the impact should maintain the same order for all integration periods, despite presenting different impact values for each of the different periods of integration.

## 4. Experiments

In this section, we present the conducted experiments to evaluate the ANE impact on the $L_{Aeq}$ computation. First, the main characteristics of the considered real-life audio dataset are described. Next, the individual analysis of all ANE impact is detailed for three different integration times: 1, 5 and 15 min. The minimum integration time has been set to 1 min since it is the shortest period necessary to include the longest ANE. The 5 min interval is chosen according to the DYNAMAP project specifications [13], and the 15 min span is also considered as the maximum time interval defined by the shortest period of a recording location. Finally, the aggregated impact of he ANEs on the $L_{Aeq}$ is also computed for the 5 min and the 15 min integration time, in order to evaluate a real-life situation, where several ANEs usually occur in a time integration period. Literature suggests that estimation of daily indicators can be extrapolated from short time spans when road traffic is the studied source. In [36], the study of the minimum measurement time interval is conducted for several noise sources including road traffic. However, in our project, continuous measurements are provided; thus, it is not necessary to use short time periods to obtain the hourly and daily indicators.

### 4.1. Real-Life Dataset

The dataset used for this experiments was recorded by means of two measuring campaigns conducted in the two pilot areas of the DYNAMAP project. Specifically, the recordings were conducted on the A90 motorway surrounding Rome as a suburban area, and the district 9 of Milan as an urban area. The used real-life dataset contains 4 h and 44 min of suburban audio and 4 h and 24 min of urban recordings. The former consists of five locations captured in the ring-road of Rome, where the shorter recording is 50 min and the longest is 1 h and 35 min. In addition, the latter consists of environmental sounds from 12 different locations within the Milan urban area, where the minimum audio duration is 15 min and the maximum 47 min. For more details about the real-life dataset and the urban and suburban recording campaign, the reader is referred to [15].

In the suburban scenario, 261 anomalous events were recorded with a total duration of 543 s, while, in the urban scenario, 711 ANEs were labelled with a total duration of 1932 s. In the suburban area, the ANEs occupy the 3% of the total recorded time, while, in the urban scenario, the presence of ANEs increase to 12%. To classify the ANE diversity in subcategories, these labels were used as defined in [15]:

*airp*: airplanes, *musi*: music coming from cars, *bike*: bikes, *peop*: people talking, *bird*: birdsong, *sire*: sirens, *brak*: vehicle brakes, *stru*: structure sounds and vibrations, *busd*: bus or tram doors, *thun*: thunders, *chai*: chains (e.g., from bikes), *tram*: tramway pass-by, *dog*: dog barks, *tran*: train pass-by, *door*: house or vehicle doors, *trck*: hitch and towing system sounds of heavy-load vehicles, *horn*: vehicle horns, *wind*: noise of wind or leaf movements and *mega*: public address system.

Regarding the SNR and duration measures of the ANEs, there is a variety of options recorded. On the one hand, the duration of the ANEs differ from 40 ms (a door recorded in Rome) to 53 s (music sound heard in Milan), with 0.6 s being the average duration value of the corpus. On the other hand, the SNR of the ANEs differ from −9.5 dB (a 3-s conversation recorded in Milan) to 27.3 dB (an extremely short door sound of Milan), becoming the average SNR value of all ANEs, 1.2 dB. However, since the study aims to evaluate the additive impact of the ANEs, we remove from the analysis those ANEs presenting subtracting impact, which, in turn, allows the use of the logarithmic representation, more illustrative to observe the impact of each individual ANE. However, as different integration times may affect the impact calculation, the number of positive-impact ANEs vary from 258 to 347 in Milan and from 129 to 141 in Rome.

### 4.2. Individual ANE Impact on the $L_{Aeq}$ Computation

**Figure 3:**
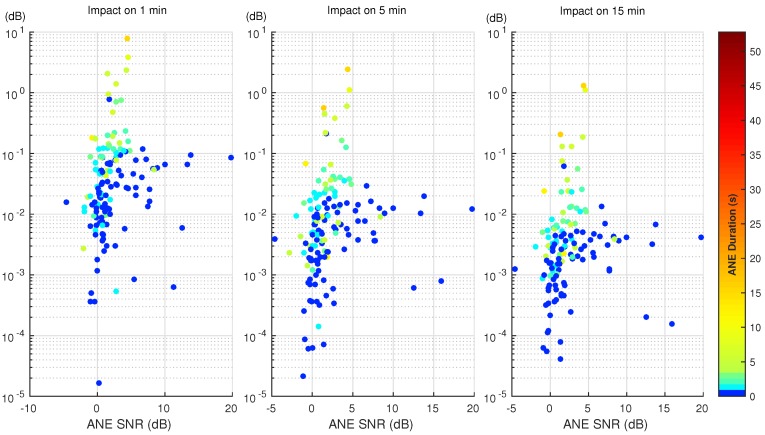
Impact of individual ANEs on the $L_{Aeq}$ value for the 1, 5 and 15 min integration times in the suburban scenario.

**Figure 4:**
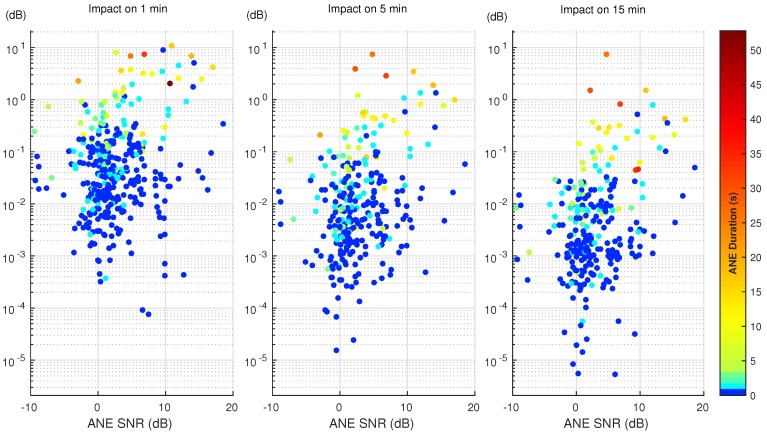
Impact of individual ANEs on the $L_{Aeq}$ value for the 1, 5 and 15 min integration times in the urban scenario.

The first part of the analysis studies and evaluates the impact of each ANE of the dataset on the $L_{Aeq}$ for a particular integration time. To that effect, the methodology explained in Section 3 is applied for each ANE belonging to the urban and suburban corpus, Milan and Rome, respectively. Both SNR and duration are calculated and the impact is measured as the individual contribution of each ANE to the given integration period. Figure 3 and Figure 4 show the impact of each ANE in a scatter plot in both urban and suburban scenarios, respectively. The *x*-axis represents the SNR of the ANE according to the previously-defined calculation (see Section 3.1). The color bar depicts the duration of the event in seconds, and the colormap has been modified to distinguish easily the shorter ANEs. Finally, the *y*-axis displays the contribution that this particular ANE has in the 1, 5 and 15 min computation of $L_{Aeq}$ in a logarithmic scale, which depicts the distribution of the impact by decades.

In both Figure 3 and Figure 4, the ANEs present a similar pattern, where high-impact ANEs have at least a long duration or a high SNR. In addition, the reader may observe that the higher the integration time, the lower is the impact of the ANEs. This is because of the nature of the impact calculation, the individual contribution decreasing in longer integration times as the duration of the ANE remains the same, and the background noise has more presence. Thus, more impact ANEs are found in the 1-min integration time.

In order to quantify the contribution, these ANEs could have in the whole $L_{Aeq}$ computation, three impact regions have been defined, discerning between those ANEs affecting the total noise level and those which not. The low-impact region is defined between 0 and 0.5 dB, the medium-impact region from 0.5 to 2 dB and, the high-impact region comprises the ANEs that surpass the 2 dB threshold. Following the defined regions, both suburban and urban scenarios are described in detail below.

In the suburban scenario, Figure 3, four ANEs surpass the 2 dB limit when analyzing the impact in 1 min, and only one surpasses the same limit in the 5-min integration time. In the 15-min integration time, no ANEs are above the 2 dB threshold. Another range can be defined from 0.5 dB and the 2 dB limit, embracing the medium-impact ANEs. In the 1-min integration time, five ANEs are comprised in this region, while three belong to the same region in a 5-min span. Only two ANEs belong to the medium-range impact region in the 15-min integration time, both being above 1 dB.

Regarding the urban scenario, Figure 4, which has a 12% presence of ANEs in comparison to the 3% of the previous scenario, more ANEs are recorded to have a higher impact. In the 1-min integration time, 18 ANEs are above the 2 dB limit, the value decreases to four in the 5-min case and only one ANE is higher than the 2 dB threshold in the 15-min span. When analyzing the medium-impact range, 22 ANEs are comprised between 0.5 dB and 2 dB in the 1-min integration time, 11 ANEs in the 5-min case and only five in the 15-min integration period.

**Figure 5:**
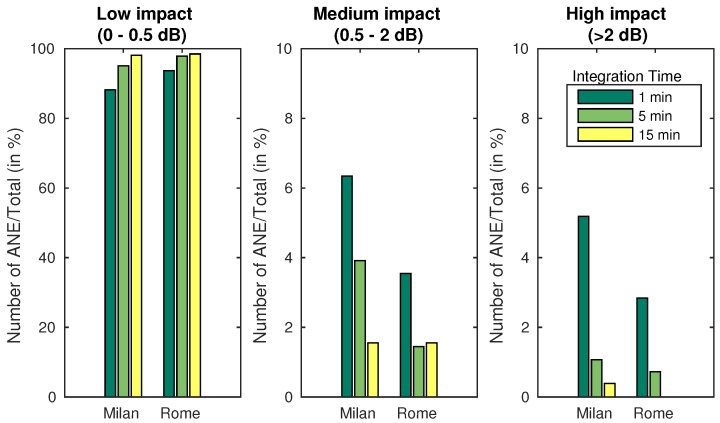
Distribution of the individual ANEs according to their impact on the $L_{Aeq}$ computation for the urban (Milan) and suburban (Rome) scenarios.

Furthermore, a representation of the ANEs belonging to each region is presented in Figure 5. The reader may observe the classification of the ANEs in three defined regions of impact, represented as a percentage in respect to the total of the correspondent scenario and integration time.

As seen in Figure 3 and Figure 4, and summarized briefly in Figure 5, few ANEs may potentially affect the $L_{Aeq}$ computation of a certain integration time. A difference between the two scenarios can be observed, as the number of high and medium impact ANEs recorded in the urban area of Milan is larger (proportionally to the total captured ANEs). Furthermore, the integration time affects deeply on the impact of the ANE; most ANEs have a great impact if measured in a shorter integration time, while the the impact decreases as the integration time increases, so more presence of background noise is also included in the $L_{Aeq}$ evaluation.

However, the defined integration time is 5 min [13] for the requirements of the DYNAMAP project, yielding to a detailed analysis of this particular integration time. In Table 1, the ANEs belonging to high and medium impact regions, given a 5-min integration time, are described.

The reader may observe that the urban scenario presents more significant ANEs with higher impact on the $L_{Aeq}$ computation than the suburban one, trains, sirens and truck pass-bys being the ones attaining the highest impact. The duration of the ANEs varies from 16 to 32 s and their SNR varies from 2.2 to 11 dB. The ANE with the highest impact is a train recorded in the urban scenario, but it is not the longest event, since the maximum duration is 53 s. It has a 4.8 dB SNR, which is below the 27.3 dB of the highest ANE’s SNR. The ANEs comprised in the medium impact region belong to sirens, in the suburban scenario and to horns, trains and tramway pass-bys. The SNR of the sirens captured in Rome, i.e., suburban scenario, differs from 1.5 to 4.5 dB and the ANEs last from 6 to 16 s. However, the typology of the ANEs changes in the urban scenario of Milan, as the horns are shorter than 2 s and have a SNR above 9 dB. The train and tramway sounds last from 6 to 18 s and comprise a great variety of SNRs, from 2.6 to 15.4 dB.

In addition, the duration and the SNR have an impact on the $L_{Aeq}$ calculation. In both the suburban and the urban scenario, the pattern of the ANEs is similar and the basic difference is that the contribution of the ANE is higher in shorter integration times and higher in longer periods. The suburban scenario in Table 1 presents only one siren with 4.4 dB of SNR and duration of 16 s, generating an impact of 2.4 dB after 5 min $L_{Aeq}$ evaluation. The urban scenario presents more variate types of ANE and longer duration, as the siren with only 2.2 dB of SNR but lasting 30 s, with an impact of 3.9 dB, a high value in the range of the results of our analysis. The urban scenario propitiates the occurrences of ANE with higher SNR (the measuring point is usually closer to the noise source than in a suburban environment), and urban environments present more actors living in the street (e.g., people, animals, sounds coming from houses).

### 4.3. Aggregated Impact of All ANEs

From the previous section, it can be observed that several individual ANEs should be removed from the road traffic noise level calculation due to their high impact—more than 2 dB—over the $L_{Aeq}$ value. However, this individual analysis should be verified to address what has been observed in real-life acoustic data, where several ANEs can occur within a predefined integration time, and depending on the density of ANEs, a series of low-impact and medium-impact events could also exceed the 2 dB threshold. In this consideration, ANEs with high SNR and small duration, or ANEs with moderate SNR and longer duration could become a key component of this aggregated impact value (see Figure 1 for more detail). Next, several examples showing the aggregated impact of ANEs on $L_{A5min}$ are included as a previous step to the 15-min impact study.

**Figure 6:**
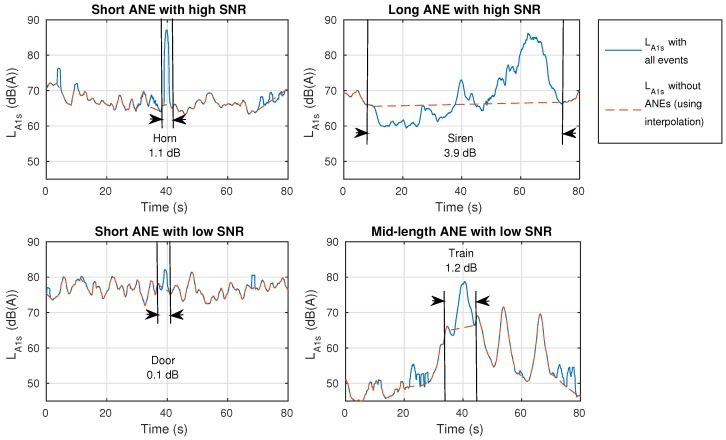
Four examples of ANEs showing different individual impact on the $L_{A5min}$ computation, considering a reference integration time of 1 s for resolution purposes.

Four particular ANEs belonging to urban and suburban scenarios are depicted in Figure 6, where a representative combination of duration and SNR parameters have been chosen to illustrate the low-, medium- and high-impacts in the $L_{A5min}$ calculation. For illustrative purposes and to improve the comparative, the *x*-axis has been established to 80 s. The bottom left ANE belongs to a door sound and has nearly no impact on the $L_{A5min}$ measurement. Bottom right example corresponds to a train pass-by with long duration and moderate SNR; it represents an individual impact of 1.2 dB, which is under the 2 dB threshold, but together with other occurring ANEs in the same integration time can lead the global impact to a value over 2 dB. Something similar happens with the top left example, which contains a horn with a short duration but a high SNR, whose individual impact on the $L_{A5min}$ sums up to 1.1 dB. Finally, the top right example is a siren with an impact of 3.9 dB on the $L_{A5min}$ value, which could be even higher if other ANEs were found in the surrounding 5-min piece of sound, and it could also have a relevant impact for larger integration times.

**Figure 7:**
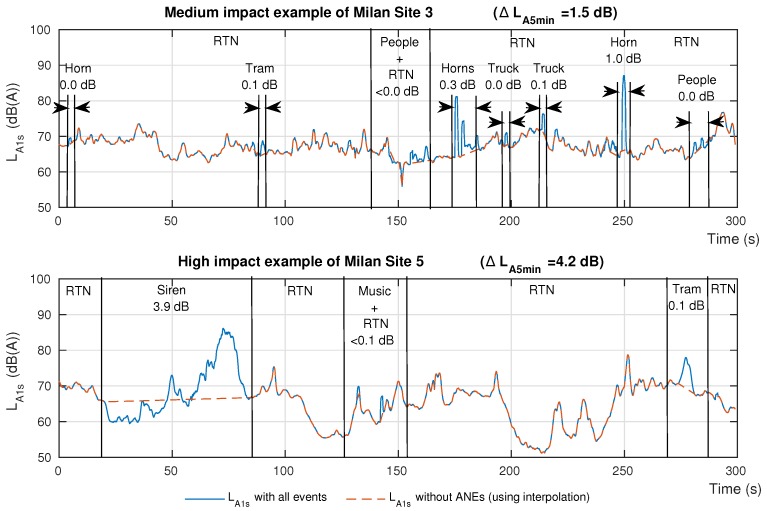
Joint impact of all the existing ANEs in the $L_{A5min}$ measurement for two different recording locations in the urban environment.

In Figure 7, the reader may observe a $L_{A5min}$ of two different recordings in the urban area. The upper example contains several horns, two truck hitching system noises, two conversations near the microphone and a tramway pass-by. From these ANEs, the ones biasing the $L_{A5min}$ more significantly are two horns of 0.3 and 1.0 dB each. Once the impact is evaluated, considering all of the involved ANEs, it rises to 1.5 dB, becoming an aggregated set of ANEs with a medium-impact on the $L_{A5min}$ computation. This is an example of why the medium-impact ANEs should also be removed from the data to compute the $L_{Aeq}$.

Moreover, in Figure 7, the lower example belongs to a 5 min fragment that contains a siren yielding a high individual $L_{Aeq}$ impact (around 3.9 dB). The joint impact of the three ANEs existing in this example sums up to 4.1 dB of impact over the total $L_{A5min}$, which is clearly over the threshold of 2 dB.

Below, the aggregated impact is also analyzed in the 15-min time span as the maximum integration time including all recordings. In Table 2, the highest impact in terms of $\Delta L_{A15min}$ within the Milan area are shown, including a brief description of the contained ANEs. A high presence of tramway and train pass-bys are recorded in Site 4 and Site 5. In addition, several high-SNR horns and hitching systems have been captured in Site 3, whose ANEs represent an impact of 1.9 dB on the $L_{A15min}$ calculation. In Rome, the maximum impact calculation in the 15-min interval is 0.2 dB, a non-relevant contribution to the $L_{Aeq}$ calculation.

It is worth mentioning that several ANEs have proven to have a significant impact on the $L_{Aeq}$ value for all the considered integration times. For instance, the siren depicted in the top-right example of Figure 6 belongs to the urban scenario and results as a high-impact individual ANE in Figure 4 and in Table 1 (with an $\Delta L_{A15min}=3.9$ dB). Moreover, the same siren is the main one responsible for the $\Delta L_{A15min}=1.9$ dB impact of the second row of Table 2, from this table, the reader may also notice that the second and the third examples show more heterogeneity in the variety of occasionally aggregated ANEs, not as in the first and the last rows, where the impact on the $L_{A15min}$ value is caused by the same typology of ANEs, i.e., trains and tramways, respectively.

As a general conclusion, the aforementioned examples show how the presence of low- and/or medium-impact ANEs can modify the aggregated impact calculation regardless of the homogeneity or heterogeneity found in the time interval, thus, all ANEs that could bias the equivalent noise level of the map must be taken into account. The high-impact ANEs affect greatly the $L_{Aeq}$ and must be considered, but also intervals with several medium-impact ANEs can be biased. Hence, both medium- and high-impact ANEs must be detected and identified if public health studies have to be derived from the noise map.

## 5. Discussion

The performed analyses on real-life urban and suburban acoustic data have led us to conclude that the impact of the ANEs on the calculation of the $L_{Aeq}$ value can become very significant (higher than 2 dB), making the ANE removal mandatory for deriving a reliable noise map from RTN. In the following sections, we discuss several questions that can be derived from these results.

### 5.1. Evaluation of the Impact of ANEs on $L_{Aeq}$ Considering Real-Life Data

In this paper, the impact on the computation of $L_{Aeq}$ has been evaluated for every individual ANE over three different integration times using both urban and suburban acoustic data. The results show that the pattern of the results of impact for most ANEs remain the same—the impact of each ANE remains sorted with the same order—despite the final value of impact decreasing as the integration time increases (see the comparisons in Figure 3 and Figure 4). The individual ANE analysis presents interesting results to be discussed using both SNR and duration of the ANE; the results in Section 4 present several ANEs with critical impact, usually related to high SNR and long duration. Several details are also presented about the medium impact ANEs, which, despite presenting impacts from 0.5 dB to 2 dB, can not be neglected in terms of this study. Nevertheless, the other scenarios obtained from the values of SNR and duration have to be studied deeply.

After the individual analysis of the impact of ANEs on the equivalent noise level computation, the work has also taken into account that dynamic acoustic mapping in *real-life* conditions face a more complex operating scenario. Within a predefined integration time, several ANEs can occur in real-life data, so the ANE impact has to be evaluated in an aggregated way. Figure 7 illustrated a couple of examples where the final aggregated impact is significantly higher than the individual impact of each ANE by itself in the $L_{A5min}$ , following the DYNAMAP project specifications. In this scenario, the aforementioned medium individual impact of a particular ANE can lead to a significant impact on $L_{Aeq}$ if surrounded by other ANEs entailing low and/or moderated individual impacts. In this sense, the medium-impact ANE group has been defined in the range [0.5, 2] dB, but all positive impact ANEs should be taken into account in terms of aggregated results in an integration time period.

Another relevant result of the individual ANE analysis is the presence of ANEs with negative SNR. As detailed in Section 3.1, the SNR is evaluated taking into account a particular ANE in relation to the surrounding RTN signal level; in certain cases, the RTN noise decreases as the ANE occurs, so a negative SNR is obtained. After the individual study, it can be concluded that only events with positive SNR should be removed from the $L_{Aeq}$ computation, otherwise, we could face an unrealistic situation of a negative impact on the $L_{Aeq}$: the equivalent level could be lower taking into account the ANEs. In this case, the closer location to the real $L_{Aeq}$ value is to consider the ANEs to perform the integration. The reason is due to the RTN or the background noise equivalent level during an ANE, which is lower, and this value should be considered as the most suitable in the total $L_{Aeq}$ value for the period.

Finally, the 15-min joint study shows that many cases exist in the urban area where the labeled ANEs bias the $\Delta L_{A15min}$ calculation in more than 1 dB and, in one case, in almost 3 dB. Thus, a presence of medium- and high-impact ANEs could distort the noise map also in larger time periods if needed in any evaluation.

### 5.2. Impact of the ANEs on the Population

The results presented in this paper detail a clear impact of the ANEs over the evaluation of the $L_{Aeq}$ value. However, a substantial impact in terms of dB may not correspond to a proportional annoyance to the people hearing these anomalous noise events. Many studies have been conducted detailing the adverse effects of traffic noise on health, but, to complete the characterization of the ANEs in the streets of an urban and suburban environment, we should test whether the impact on the quantitative measures used in this paper are correlated with the annoyance of the affected population in terms of their subjective perception. In addition, analyzing the acoustic measure every 5 min allows for informing the authorities and population in a maximum delay of 5 min. Several key questions still remain open. Is a siren more annoying than a train, having both the same acoustic impact? Or maybe the duration of the ANEs is a key factor in the disturbance? Or the salience in comparison with the background noise? The next steps of this study pretend to make way towards subjective analysis of the noise impact and, eventually, to contribute to a method of determining the impact of certain sound events to the citizens taking into account the effects of ANEs in these kinds of studies.

## 6. Conclusions

This work has introduced an analysis methodology to evaluate the impact of the ANEs on the $L_{Aeq}$ computation for different integration times in both real-life urban and suburban areas. The conducted experiments allow for concluding that any automatic approach to noise monitoring and control should take into account the impact of anomalous noise events on the road traffic noise $L_{Aeq}$ computation. To that effect, both SNR and duration have been proven to be key variables to determine the impact of either individual or aggregated ANEs on the equivalent noise level for a predefined integration time. In this sense, the results show that the impact of individual ANEs can be substantial, especially in an urban environment, but when the study is widened by taking into account several ANEs occurring within the same period simultaneously, the analysis shows that even those ANEs attaining individual low- or medium-impact may contribute to surpass the 2 dB defined threshold that determines a substantial change in the $L_{Aeq}$ evaluation. For this purpose, a deeper analysis on the evaluation of the $L_{Aeq}$ value when considering all positive impact ANEs for a certain integration period will be conducted in the future. The goal is to determine the minimum values of SNR and duration for the ANEs to suggest its removal and to find the minimum number of ANEs needed to have an impact of more than the threshold of 2 dB.

Another conclusion obtained from this study is that negative SNR can be occasionally obtained when parametrizing the ANE database. Due to the relative nature of the SNR parameter definition, these results could be obtained when the ANE is quieter than the surrounding background noise. The detailed analysis of this kind of situations is left for future works.

The duration and the SNR have been proven to be key values to model the nuisance that an ANE causes to the neighbourhood in real-time (e.g., every 5 min as agreed within the DYNAMAP project). On the one hand, when working in short time spans, all ANEs non-related to the source under study, traffic noise in this case, should be removed from the noise map computation to avoid biasing the results. However, on the other hand, it allows for making fast decisions to inform the exposed citizens or the local authorities if unusual noise levels are recorded.

We would like to close this work with a question. Is the impact of the ANEs on $L_{Aeq}$ a good measure for annoyance in the neighbourhood? We know from literature that the equivalent noise level is a good indicator to find a relationship between high noise measurements and several illnesses. The study of the degree of annoyance in the citizens considering anomalous noise events would allow us to study if the impact, the SNR and/or the duration, are key values to modelling the nuisance that an ANE causes to the neighbourhood. This research will entail the next step of our investigations.

## Figures and Tables

**Table 1:** Anomalous Noise Events (ANEs) of the high- and medium-impact regions from the suburban (Rome) and urban (Milan) scenarios.

Region	Scenario	Type	Signal-to-Noise Ratio (dB)	Duration (s)	$\Delta L_{A5\min}$ (dB)
High Impact (>2dB)	Suburban	Siren	4.4	16	2.4
	Urban	Train	4.8	26	7.4
		Siren	2.2	50	3.9
		Truck	11.0	17	3.5
		Train	6.9	32	2.8
Medium Impact (0.5–2 dB)	Suburban	Siren	4.5	9	1.1
		Siren	4.3	6	0.6
		Siren	1.5	16	0.6
	Urban	Train	13.8	18	1.9
		Horn	12.0	2	1.4
		Horn	14.3	1	1.4
		Train	2.6	7	1.2
		Horn	9.6	1	1.1
		Tramway	17.1	15	1.0
		Tramway	12.0	10	0.8
		Tramway	15.4	9	0.8
		Tramway	3.8	6	0.6
		Tramway	3.5	15	0.6
		Horn	9.6	1	0.6

**Table 2:** Detail of the highest impact of aggregated ANEs on $L_{A15min}$ within the Milan urban area for different recording sites.

Scenario	Location	$\Delta L_{A15\min}$ (dB)	Description
Milan	Site 4	2.7	Train pass-bys of 140 s in total.
	Site 5	1.9	Tramway pass-bys of 40 s in total and a 50-s high-SNR siren.
	Site 3	1.9	Many high-SNR horns and three high-impact hitching system noises.
	Site 5	1.3	Tramway pass-bys of 80 s in total.

## Abbreviations

The following abbreviations are used in this manuscript:

ANE	Anomalous Noise Event
CNOSSOS-EU	Common Noise Assessment Methods in Europe
DALYs	Disability-Adjusted Life-Years
END	Environmental Noise Directive
RTN	Road Traffic Noise
SNR	Signal-to-Noise Ratio
WASN	Wireless Acoustic Sensor Networks
WHO	World Health Organization
